# Harmful lifestyles' clustering among sexually active in-school adolescents in Zambia

**DOI:** 10.1186/1471-2431-8-6

**Published:** 2008-02-11

**Authors:** Seter Siziya, Adamson S Muula, Lawrence N Kazembe, Emmanuel Rudatsikira

**Affiliations:** 1 Department of Community Medicine, University of Zambia, School of Medicine, Lusaka, Zambia; 2 Department of Community Health, University of Malawi, College of Medicine, Blantyre, Malawi; 3 Department of Mathematical Sciences, University of Malawi, Chancellor College, Zomba, Malawi; 4 Departments of Epidemiology and Biostatistics and Global Health, Loma Linda University, School of Public Health, Loma Linda, California, USA

## Abstract

**Background:**

HIV is a leading cause of morbidity and mortality in Zambia. Like many other African nations with high HIV burden, heterosexual intercourse is the commonest mode of HIV spread. The estimation of prevalence   and factors associated with sexual intercourse among in-school   adolescents has potential to inform public health interventions aimed   at reducing the burden of sex-related diseases in Zambia.

**Methods:**

We carried out secondary analysis of the Zambia Global School-Based Health Survey (GSHS) 2004; a cross sectional survey that aims to study health-related behaviors among in-school adolescents. We estimated frequencies of relevant socio-demographic variables. The associations between   selected explanatory variables and self-reported history of sexual   intercourse within the last 12 months were assessed using logistic   regression analysis.

**Results:**

Data from 2136 in-school adolescents who participated in the Zambia Global School-Based Health Survey of 2004 were available for analysis. Out of these respondents, 13.4% reported that they had sexual intercourse in the past 12 months prior to the survey; 16.4% and 9.7% among males and females respectively. In multivariable logistic regression analysis, with age less than 15 years as the referent the adjusted odds ratio (AOR) of having engaged in sexual intercourse in adolescents of age 15 years, and those aged 16 years or more were 1.06 (95% CI 1.03–1.10) and 1.74 (95% 1.70–1.79) respectively. Compared to adolescents who had no close friends, adolescents who had one close friend were more likely to have had sexual intercourse, AOR = 1.28 (95% CI 1.24–1.32). Compared to adolescents who were not supervised by their parents, adolescents who were rarely or sometimes supervised by their parents were likely to have had sexual intercourse, and adolescents who were most of the time/always supervised by their parents were less likely to have had sexual intercourse; AORs 1.26 (95% CI 1.23–1.26) and 0.92 (95% CI 0.90–0.95) respectively. Compared to adolescents who did not smoke dagga, adolescents who smoked dagga 1 or 2 times, and those who smoked dagga 3 or more times in their lifetime were 70% and 25% more likely to have had sexual intercourse, respectively. Adolescents who drank alcohol in 1 or 2 days, and those who took alcohol in 3 or more days in a month preceding the survey were 12% and 9% more likely to have had sexual intercourse, respectively, compared to adolescents who did not drink alcohol in the 30 days prior to the survey. Furthermore, adolescents who had been drunk 1 or 2 times, and who had been drunk 3 or more times in a life time were 14% and 13% more likely to have had sexual intercourse compared to those who have never been drunk in their lifetime.

**Conclusion:**

We identified a constellation of potentially harmful behaviours among adolescents in Zambia. Public health interventions aimed at reducing prevalence of sexual intercourse may be designed and implemented in a broader sense having recognized that sexually active adolescents may also be exposed to other problem behaviours.

## Background

Southern Africa is the region of the world that has the highest prevalence of HIV infection in the world. According to the Zambia Demographic and Health Survey 2001–2, HIV prevalence among 15 to 49 year olds in Zambia was estimated at 15.6% [1]. Although HIV prevalence is that high, there is some glimmer of hope in the country. Sandoy et al have reported on the decline in sexual risk behaviours among 15 to 24 year olds in Zambia between 1995 and 2003 [2]. These authors concluded that fewer sexual partners and condom use for both sexes, and delayed child-bearing were among the core factors that were associated with the decline in the HIV prevalence.

As adolescence is a critical period for physical, social, and emotional development, the possibility of adolescents engaging in sexual intercourse is an important social and public health consideration. Sexual intercourse among adolescents is a risk factor for teen pregnancy, clandestine abortions, sexually transmitted infection, school dropout, and HIV [3,4]. Adolescents who engage in sexual intercourse may also be exposed to harmful lifestyles such as use of illicit drugs, alcohol, and cigarette smoking [5,6]. The national HIV public health interventions in Zambia among adolescents have largely been geared towards sexual abstinence, mostly because of significant resources and influence from the US President's Emergency Plan for AIDS Relief (PEPFAR) that has particular emphasis on abstinence [7]. This emphasis in prevention interventions may be limited as it may not cater for adolescents with diverse experiences.

In order to inform the design and implementation of public health intervention aimed to delay sexual debut and promote "safer sex" when it occurs, there is need for the identification of variables that are associated with teen sexual intercourse. We therefore carried out secondary analysis of data from the 2004 Zambia Global School-Based Health Survey (GSHS) to identity factors associated with history of sexual intercourse among in-school adolescents in Zambia.

## Methods

### Primary and secondary education in Zambia

The Zambia education system consists of 7 years primary education (Grades 1 to 7), 2 years of junior secondary education (Grades 8 and 9), and senior secondary school (Grades 10 to 12). Although some children are enrolled into primary school when they are aged below 7 years, the official age at enrolment in primary school is 7 years. The school system is undergoing   restructuring from the 3-level system (Primary, Junior and Secondary) to 2-level:Basic education (Grades 1–9); and High school (Grades 10–12). Not all children of school   going age are enrolled in schools. In 2002, 85% of boys and 78% of girls were enrolled at primary level (Ministry of Education, 2002 Education Statistical Bulletin, Directorate of Planning and Information [unpublished]). Out of 4558 primary schools in Zambia in 2002, 94% were government schools, 5% private schools, 1% government assisted schools, and the rest were other types of schools. The number of secondary schools in the same year was 335. The percentages for boys and girls enrolled in secondary schools were 15% and 11%, respectively of the age-eligible population [Strategic Plan 2003–2007, Ministry of Education, Zambia].

### Study design and participant recruitment in the GSHS

Our study involved secondary analysis of data from the Zambia Global School-Based Health Survey (GSHS) conducted in 2004. The GSHS was developed by the World Health Organization (WHO) in collaboration with UNICEF, UNESCO, and UNAIDS with technical assistance from the Centres for Diseases Control and Prevention (CDC), Atlanta, Georgia, United States. The GSHS aims to provide data on health and social behaviours primarily among in-school adolescents aged 13–15 years.

The GSHS uses a two-stage probability sampling technique. In the first stage of sampling, primary sampling units are schools that are selected with a probability proportional to their enrolment size. In Zambia 50 out of 4621 government schools were selected from all the nine provinces. However, 47 (94%) schools participated in the survey. In the second step, a systematic sample of classes in the selected school is obtained. All students in the selected classes are eligible to participate. Most of the children of age 13–15 years are in Grades 7–9 in Zambia. However all children in Grades 7–10 were requested to participate in the study. Out of 3021 adolescents who were in Grades 7–10, 2257 participated in the survey, giving a response rate of 75%. However data from 2136 participants were available for analysis in this report. A questionnaire was anonymously completed by the students. Completion of questionnaires occurred within one class period, and 12 trained research assistants supervised the process.

### Ethical considerations

Ethical review and permission was obtained both the Ministry of Health and Education provided permission for the study to be conducted. Students invited to participate in the study were informed that they were free not to participate and that they were free not to answer any questions on the questionnaire. Study participants self-completed without any personal identifiers. At each of the study sites, the head teacher provided permission for data collection.

### Data analysis

Data that we used for this study included variables on having had sexual intercourse within the last 12 months, marijuana (dagga) smoking, alcohol use, gender, having ever been drunk, number of close friends they had and adolescents' own assessment of parental supervision. Some of the questions asked were: During the past 12 months, have you had sexual intercourse? During your life, how many times have you used dagga? Other questions were: How many close friends do you have? During your life, how many times did you drink so much alcohol that you were really drunk? During the past 30 days, on how many days did you have at least one drink containing alcohol?

Data analysis was performed using SPSS version 14.0 software. A weighting factor was used in the analysis to reflect the likelihood of sampling each student and to reduce bias by compensating for differing patterns of non-response.

We obtained frequencies as estimation of prevalence. We conducted backward logistic regression analysis to estimate associations between relevant predictor variables and sexual intercourse within the last 12 months. Relevant predictor or explanatory variables obtained from the literature were: age; sex or gender; alcohol use, having ever been drunk, parental supervision and social networking with friends [8-18]. Reddy et al [8] have reported that female gender was associated with lower likelihood of alcohol and marijuana use among adolescents in South Africa. However, in the United States, while females were associated with lower marijuana use, they were not less likely to use alcohol compared to males. Delva et al have also reported higher likelihood of marijuana or dagga use among males compared to females [9]. Kliewer and Murrelle [10] have reported that adolescent marijuana use was associated with alcohol and other substance use among adolescents in Central America. Thompson and Auslander [11] and White et al [12] have reported that close parental supervision is associated with lower marijuana and lower alcohol use.

We report unadjusted odds ratios for selected predictor variables while considering having engaged in sexual intercourse in the last 12 months as a dependent variable. We also report results for multivariate analysis (adjusted odds ratios) for the factors found significantly associated with the outcome in the bivariate analysis.

## Results

Data from 2136 in-school adolescents who participated in the Zambia Global School-Based Health Survey in 2004 was available for analysis. Of these respondents, 182 reported having had sexual intercourse in the past 12 months, 1213 indicated that they had no sexual intercourse in the preceding 12 months to the survey, and 741 did not respond to the question whether they had sexual intercourse in the past 12 months. Among the participants who responded to the question, 13.4% reported that they had sexual intercourse in the past 12 months prior to the survey. The prevalence of sexual intercourse among males was 16.4%, and 9.7% among females. The prevalence of sexual intercourse among adolescents of age less than 15 years was 9.5%.

Table 1 shows the distributions of the selected explanatory variables between respondents who had sexual intercourse in the 12 months prior to the survey and those who had no sexual intercourse in the same period. In bivariate analyses, age, male gender, having smoked dagga in a life time, having close friends, having taken alcohol, and having been drunk were positively associated with having had sexual intercourse. Meanwhile, parental supervision was protective against having sexual intercourse

**Table 1:** Associations of selected explanatory variables and having had sexual intercourse 12 months prior to the   survey in Zambia.

**Factor**	**Had sexual intercourse**		**Unadjusted OR (95% CI)**
	Yes, N* (%)*	No, N* (%)**	
**Age**			
<15	52 (33.9)	500 (48.1)	1
15	41 (24.1)	293 (22.6)	1.04 (1.02–1.06)
16+	78 (42.0)	393 (29.3)	1.40 (1.37–1.42)
			
**Sex**			
Male	110 (66.3)	541 (51.8)	1
Female	63 (33.7)	624 (48.2)	0.74 (0.73–0.75)
			
**Number of close friends**			
0	15 (8.2)	182 (15.7)	1
1	69 (36.9)	356 (28.0)	1.50 (1.47–1.54)
2 +	101 (54.9)	655 (56.2)	1.12 (110–114)
			
**Parental supervision**			
Never	35 (23.7)	207 (19.5)	1
Rarely or sometimes	70 (40.1)	453 (42.9)	1.07 (1.05–1.09)
Most of the times or always	48 (30.2)	398 (37.6)	0.82 (0.81–0.84)
			
**Days drank alcohol past 30 days**			
0	60 (44.3)	563 (67.0)	1
1 to 2	24 (22.1)	98 (13.2)	1.36 (1.33–1.39)
3+	38 (33.5)	140 (19.8)	1.37 (1.34–1.41)
			
**Number of time ever used marijuana**			
0	95 (50.6)	870 (72.9)	1
1 to 2	51 (30.8)	172 (15.3)	1.54 (1.51–1.57)
3+	30 (18.7)	129 (11.8)	1.21 (1.19–1.24)
			
**Number of times ever been drunk**			
0	90 (48.8)	761 (65.1)	1
1 to 2	49 (28.1)	237 (21.4)	1.10 (1.08–1.13)
3+	42 (23.1)	141 (13.5)	1.44 (1.37–1.44)

Row frequencies not adding up because of missing information

* unweighted frequencies

** weighted percents

In multivariable analysis (Table 2), the factors that were significantly associated with the outcome in bivariate analyses remained significant in multivariate analysis. Adjusted odds ratios (AORs) are reported as measures of association in the multivariable analysis. Compared to adolescents of age less than 15   years, adolescents of age 15 years, and those aged 16 years or more   were 6% (AOR = 1.06, 95% CI 1.03, 1.10) and 74% (AOR = 1.74, 95% CI 1.70, 1.79) more likely to have had sexual intercourse, respectively. Female adolescents were 34% (AOR = 0.66, 95% CI 0.64, 0.67) less likely to have had sexual intercourse compared to male adolescents. Compared to adolescents who had no close friends, adolescents who had one close friend were 28% (AOR = 1.28, 95% CI 1.24, 1.32) more likely to have had sexual intercourse. However, the odds of having had sexual intercourse were similar between adolescents who had 2 or more close friends and those who had no close friends. Compared to adolescents who were not supervised by their parents, adolescents who were rarely/sometimes supervised by their parents were 7% (AOR = 1.07, 95% CI 1.05, 1.09) more likely to have had sexual intercourse, and adolescents who were most of the time/always supervised by their parents were 18% (AOR = 0.82, 95% CI 0.81, 0.84) less likely to have had sexual intercourse.

**Table 2:** Factors in a multivariate analysis that were associated with sexual intercourse in past 12 months among in-school adolescents in Zambia

**Factor**	**Adjusted OR**
**Age**	
<15	1
15	1.06 (1.03–1.10)
16+	1.74 (1.70–1.79)
	
**Sex**	
Male	1
Female	0.66 (0.64–0.67)
	
**Number of close friends**	
0	1
1	1.28 (1.24–1.32)
2 +	0.98 (0.95–1.01)
	
**Parental supervision**	
Never	1
Rarely or sometimes	1.26 (1.23–1.29)
Most of the times or always	0.92 (0.90–0.95)
	
**Days drank alcohol past 30 days**	
0	1
1 or 2	1.12 (1.08–1.15)
3+	1.09 (1.06–1.14)
	
**Number of time ever used marijuana**	
0	1
1 or 2	1.70 (1.65–1.75)
3+	1.25 (1.20–1.29)
	
**Number of times ever been drunk**	
0	1
1 or 2	1.14 (1.11–1.18)
3+	1.13 (1.09–1.18)

Compared to adolescents who did not smoke dagga, adolescents who smoked dagga 1 or 2 times, and those who smoked dagga 3 or more times in their lifetime were 70% (AOR = 1.70, 95% CI 1.65, 1.75) and 25% (AOR = 1.25, 95% CI 1.20, 1.29) more likely to have had sexual intercourse, respectively. Adolescents who drank alcohol in 1 or 2 days, and those who took alcohol in 3 or more days in a month preceding the survey were 12% (AOR = 1.12, 95% CI 1.08, 1.15) and 9% (AOR = 1.09, 95% CI 1.06, 1.14) more likely to have had sexual intercourse, respectively, compared to adolescents who did not drink alcohol in the 30 days prior to the survey. Meanwhile, adolescents who had been drunk 1 or 2 times, and who had been drunk 3 or more times in a life time were 14% (AOR = 1.14, 95% CI 1.11, 1.18) and 13% (AOR = 1.13, 95% CI 1.09, 1.18) more likely to have had sexual intercourse.

## Discussion

We estimated the overall prevalence of sexual intercourse within the last 12 months at 16.4% and 9.7% among male and female, respectively, in-school adolescents in Zambia. Our finding that 9.4% of adolescents of age less than 15 years had sexual intercourse in the previous 12 months to the survey indicate that adolescents in Zambia have their first sexual intercourse at a young age. This is in support of the findings in the Demograhic and Health Survey of 2001–2002 that the median age at first sexual intercourse was 16.8 and 17.8 years for females and males, respectively [1]. In Zimbabwe, the median age at first sexual intercourse has been reported as 19 years for males and 18 years for females [13]. These estimates are much lower than   those that have been reported for the United States between 1991 and 2005 where adolescents in school Grades 9 to 12 had prevalence of sexual intercourse within the last 3 months exceeding 33% [14]. While we can make comparisons of prevalence between different settings, it is also important to recognize that differences may actually arise from differences in study questions. In the Zambia GSHS, students were asked: During the past 12 months, have you had sexual intercourse? Sexual intercourse was not specifically defined. In a study by Thurman et al in South Africa, study participants were asked whether they had ever engaged in sex, specifically described as full penile-vaginal penetration [15]. In the United States [13], the duration of recall was 3 months compared to 12 months in the GSHS. There may therefore be limitation in comparing studies that have a specific definition or definitions of sexual intercourse to those that did not.

The constellation of potentially harmful behaviours within an individual is cause for concern. Our study shows that adolescents who engage in sexual intercourse are also likely to have smoked dagga, used alcohol, and had been drunk. Ohene et al have reported that among Caribbean youth, being sexually active was a good marker of also being involved in other problem behaviours [16].

In Zambia, the school health program includes the following topics for Grades 7–9: love sex and abstinence; reproductive health; sexually transmitted infections; understanding HIV and AIDS, for Grade 7, working together safely; children's rights, growing up; and understanding gender, for Grade 8; and sexual feeling and behaviour; about pregnancy; sexually transmitted infections; understanding HIV and AIDS; and coping well with life, for Grade 9. This curriculum may require to be changed with these other harmful practices in mind. Dealing with sexual behaviour alone may not lead to healthy adolescent behaviours. On the other hand, it is possible that preventing a single harmful behaviour may also result in gains in the prevention of other associated behaviors.

It is of particular interest that adolescents who reported having close friends were more likely to have engaged in sex than those without close friends. It is possible that those adolescents who have friends may use this pool of friends as source of sexual partners. It is also plausible that having had sex may make one popular and be attracted to new friends. In a study of US adolescents, Brady and Halpern-Felsher [17] reported that some adolescents experienced popularity in the immediate period following sexual intercourse.

In our study, males were more likely to have reported sexual intercourse than females (16.4% versus 9.7%). Van Duynhoven et al have reported that females were more likely to report more reliable sexual histories than males [18]. It is not possible to determine whether such disparity in reporting may have occurred in our study. However, the fact that we identified   male gender, substance use (illicit drugs and alcohol use) and   increasing age as positively associated with history of sexual intercourse calls for consideration of these factors in efforts to   prevent sexual intercourse in adolescents or at least identify those that may require targeting for safer sex, such as condom use promotion, education on illicit drugs and prohibition of alcohol use.

We found that adolescents who reported having parental supervision were less likely to engage in sexual intercourse in bivariate analysis but quite the opposite association in multivariate analysis. Bingham and Crockett [19] have demonstrated parental support and supervision to be protective against experience with sexual intercourse. In other studies, adolescents who have parental support are more likely to use condoms and other modern family planning methods if they engage in sexual intercourse [20,21]. We cannot determine with certainty why adolescents who report sexual intercourse were also likely to report parental supervision. It is quite possible that adolescents who engage in problem behaviours, as a consequence may attract increased parental supervision.

### Limitations of the study

This study had several limitations. Firstly, the findings of this study may only be applicable to in-school adolescents. Ndyanabangi et al compared in-school and out-of-school adolescents in Uganda [22]. Out of school adolescents were more likely to have initiated sex at a younger age, were less likely to use modern contraception, and were more likely to have multiple sexual partners. Our findings may therefore not be applicable to all adolescents in Zambia. Similar results were also reported by Slonim-Nevo and Mukuka that out of school adolescent were more likely to engage in high risk sexual behaviours compared to in-school peers [23].

Secondly, data from the Global School-Based Health Survey were collected through self-completion of the questionnaire by the study participants. As study participants were requested to report behaviours that had occurred either 12 months or 30 days preceding the survey, some study participants may have failed to recall the behaviours or that the behaviours had occurred within the timeframe requested. In addition, as the data that was requested was potentially sensitive such as dagga smoking, alcohol use, and sexual intercourse among students, it is possible some students who may have engaged in such behaviours may have failed to   report them. Although the students were requested to complete the questionnaire anonymously, and the class teachers were excused during the questionnaire completion, complete accurate reporting cannot always be achieved.

Furthermore, the GSHS utilizes a cross sectional design. Due to the nature of the study design, it is not possible to ascribe causality to any of the factors identified as associated with adolescent experience of sexual intercourse.

Of the 3021 students eligible to participate in the study only 71% eventually participated. Moreover, the findings of the survey reflect the experiences of the students present in school on the day the survey questionnaire was completed. Students who were absent on the day of the survey were never followed up.

As the period of reference is relatively long, it is not possible to verify the history as can be done with very recent history with biomarkers such as prostate specific antigen in females, (when no condoms were used) [24]. Despite these limitations, our study has demonstrated that adolescent sexual intercourse is associated with other potentially harmful practices such as alcohol, dagga use, and having ever gotten drunk. Although it was not assessed as to whether adolescents have ever engaged in sex while drunk, it has been described that use of alcohol during or with sex is associated with no condom use [25,26], thus exposing the adolescent to sexually transmitted infections, including HIV. Although this link has been demonstrated in some settings, Bryan et al failed to demonstrate any association between alcohol use and non-condom use among criminally involved adolescents [27].

## Conclusion

Our results can be used as evidence that constellation of behavioural factors do exist with history of sexual intercourse among Zambian adolescents. The public health implication of interest in our study may be that it is reasonable to broaden the sexual health promotion message (abstinence, condom use) among adolescents to also include other problem behaviours such as illicit drug use, alcohol and encourage parental support [28].

## Abbreviations

CDC, Centers for Disease Control and Prevention; HIV, human immunodeficiency virus; GSHS, Global School-Based Health Survey; PEPFAR, President's Emergency Plan for AIDS Relief; UNAIDS, Joint United Nations Program on HIV/AIDS; UNICEF, United Nations Children's' Fund; UNESCO, United Nations Educational, Scientific and Cultural Organisation; WHO, World Health Organisation.

## Competing interests

The author(s) declare that they have no competing interests.

## Authors' contributions

SS: conducted the statistical analysis, interpreted the results and participated in the drafting of the manuscript.

ER: conducted repeat statistical analyses, interpreted the results and participated in the drafting of manuscript.

LM: participated in the interpretation and drafting of the manuscript.

ASM: participated in the interpretation of the results and led the drafting of the manuscript.

All authors read and approved the final draft of the manuscript.

## Pre-publication history

The pre-publication history for this paper can be accessed here:


